# The hidden factor: accounting for covariate effects in power and sample size computation for a binary trait

**DOI:** 10.1093/bioinformatics/btad139

**Published:** 2023-03-21

**Authors:** Ziang Zhang, Lei Sun

**Affiliations:** Department of Statistical Science, Faculty of Arts and Science, University of Toronto, Toronto, ON M5G 1Z5, Canada; Department of Statistical Science, Faculty of Arts and Science, University of Toronto, Toronto, ON M5G 1Z5, Canada; Division of Biostatistics, Dalla Lana School of Public Health, University of Toronto, Toronto, ON M5T 3M7, Canada

## Abstract

**Motivation:**

Accurate power and sample size estimation is crucial to the design and analysis of genetic association studies. When analyzing a binary trait via logistic regression, important covariates such as age and sex are typically included in the model. However, their effects are rarely properly considered in power or sample size computation during study planning. Unlike when analyzing a continuous trait, the power of association testing between a binary trait and a genetic variant depends, explicitly, on covariate effects, even under the assumption of gene–environment independence. Earlier work recognizes this hidden factor but the implemented methods are not flexible. We thus propose and implement a generalized method for estimating power and sample size for (discovery or replication) association studies of binary traits that (i) accommodates different types of nongenetic covariates *E*, (ii) deals with different types of *G*–*E* relationships, and (iii) is computationally efficient.

**Results:**

Extensive simulation studies show that the proposed method is accurate and computationally efficient for both prospective and retrospective sampling designs with various covariate structures. A proof-of-principle application focused on the understudied African sample in the UK Biobank data. Results show that, in contrast to studying the continuous blood pressure trait, when analyzing the binary hypertension trait ignoring covariate effects of age and sex leads to overestimated power and underestimated replication sample size.

**Availability and implementation:**

The simulated datasets can be found on the online web-page of this manuscript, and the UK Biobank application data can be accessed at https://www.ukbiobank.ac.uk. The R package SPCompute that implements the proposed method is available at CRAN. The genome-wide association studies are carried out using the software PLINK 2.0 [Purcell et al. (Plink: a tool set for whole-genome association and population-based linkage analyses. *Am J Hum Genet* 2007;81:559–75.)].

## 1 Introduction

Accurate power and sample size estimation is crucial to the design of many scientific studies, including the ubiquitous genome-wide association studies (GWAS) of complex and heritable human diseases and traits (Hong and Park 2012). It is well known that replication studies with underestimated sample sizes can result in false negatives, missing single nucleotide polymorphisms (SNPs; *G*s) that are truly associated with the phenotype of interest (*Y*) (Patil et al. 2016). Additionally, recent work (Turley et al. 2018) has shown that failure to correctly estimate power can also result in increased false positives in pleiotropy studies, where different traits are jointly analyzed and their GWAS summary statistics are aggregated.

The power and sample size calculation for a continuous trait is well established, as the phenotype–genotype association analysis is through the ordinary linear regression, regressing *Y* on *G* and important nongenetic covariates *E*s. It is then straightforward to show that the power of the corresponding genetic association test only depends on the effect size and minor allele frequency (MAF) of the SNP, sample size, and the unexplained phenotypic variance (Korte and Farlow 2013). That is, when analyzing a continuous trait, the sample size for a replication study with sufficient power is determined by the proportion of phenotypic variance explained by genetic variants, which is also called narrow-sense heritability (Mayhew and Meyre 2017; Yang et al. 2017).

In contrast, power calculations for binary outcomes require additional considerations, as the association analysis typically uses the logistic or probit regression model (Robinson and Jewell 1991; Sjölander and Greenland 2013). Most heritability estimation methods were rigorously developed for continuous traits only (Yang et al. 2010; Weissbrod et al. 2018), and their applications to binary traits have been questioned (Golan et al. 2014). At the same time, when analyzing a binary outcome *Y*, power of analyzing an SNP *G* is affected, explicitly, by the effect size of a nongenetic covariate *E*, even if *E* is independent of *G* and/or there is no *G*x*E* interaction effect (Robinson and Jewell 1991; Pirinen et al. 2012). Therefore, accurate power and sample estimation for a binary trait-genetic association analysis must explicitly consider the presence of nongenetic covariates.

There have been several attempts in the literature to consider the general problem of power and sample size computation for logistic regression. Whittemore (1981) derived an approximation method, assuming that the disease prevalence is small and the covariates have a joint distribution of multivariate exponential. The approach of Whittemore (1981) was similarly considered by Hsieh (1989, 1998) and Novikov et al. (2010). Based on the asymptotic power approximation of the score or likelihood ratio test under local alternatives, Self et al. (1992) and Self and Mauritsen (1988) proposed an alternative approach that accommodates several categorical covariates with finite configurations, which was then extended by Shieh (2000) to allow for one categorical covariate with infinite configurations.

For genetic association studies, Quanto is the most commonly used software in practice, implementing the method of Gauderman (2002a,b). The method uses the expected value of a likelihood ratio test (LRT) statistic and accommodates both continuous and categorical *E*s for power analysis of *G*x*E* interaction. However, the approach of Gauderman (2002b) implicitly assumes that *G* and *E* are independent of each other, which may not hold in practice for complex diseases (Plomin et al. 1977; Scarr and McCartney 1983; Knafo and Jaffee 2013; Zhu et al. 2018; Namjou et al. 2019). Further, the implemented software Quanto does not accommodate the presence of *E* unless the power computation is for *G*x*E* interaction analysis. That is, *E* cannot be included when the power analysis is for the main effect of *G*.

Demidenko (2007), on the other hand, advocated for the use of the Wald test to do power and sample size computation for logistic regression, and proposed a method that allows *E* and *G* to be dependent through a second-stage logistic regression model. However, the implemented web-tool (Demidenko 2008) only allows for one binary covariate, as otherwise the computation does not admit a closed-form expression.

Lyles et al. (2007) proposed a different approach to power computation for generalized linear models, based on the use of an *expanded representative* dataset. The idea of expanded representative dataset provides accurate approximation with good computational efficiency when sample size is small to medium, but the computation becomes cumbersome when the sample size is large. This is relevant as many GWAS have large sample sizes and small genetic effect sizes.

In this paper, we propose and implement a generalized method for estimating power and sample size for genetic association studies of binary traits that (i) takes into account different types of nongenetic covariates *E*, (ii) allows for different types of *G*–*E* relationship, and (iii) has good computational efficiency for large-scale studies. The utility of the proposed method is illustrated and compared with the existing methods through extensive simulation studies and an application study of the UK Biobank data (Sudlow et al. 2015; Bycroft et al. 2018). The proposed method has been implemented as a R package, SPCompute, available at CRAN.

## 2 Preliminary

### 2.1 Models

For simplicity of the notation, we assume without the loss of generality that there is only one nongenetic covariate *E*; the method implementation and the application allow for multiple *E*s. To study the relationship between a trait *Y* and an SNP *G* of interest, conditional on the nongenetic covariate *E*, we consider the following generalized linear model (glm; McCullagh and Nelder 2019), where g(·) is a link function, connecting each linear predictor *η* with the mean function of *Y*. This glm model accommodates the analyses of both continuous and binary traits. Here, we focus on binary traits, for which the logistic regression is the most commonly used model with $g(\mu )=\log(\frac{\mu }{1-\mu })$.


(1) $$g(E(Y|G,E))=g(\mu )=\beta_{0}+\beta_{G}G+\beta_{E}E=\eta ,$$

Let ***X*** be the n×3 design matrix, which has rows ${\left(1,G_{i},E_{i}\right)}_{i=1}^{n}$, where *n* is the sample size. To ease notation, we also use ***X*** to denote the observed data, and we use $\beta ={\left(\beta_{0},\beta_{G},\beta_{E}\right)}^{T}$ to denote the vector of either all regression parameters or their true values. The n×1 linear predictor vector η is then expressed as where 1,G, and ***E*** are also n×1 vectors. Following the convention in genetic association studies, the SNP genotypes, *aa*, *Aa*, and *AA*, are assumed to follow Hardy–Weinberg equilibrium (HWE; Mayo 2008), where *A* is the minor allele with MAF of *p*. Although conceptually the proposed method also accommodates nonadditive genetic effects, the default assumption is that *G* is nominal and coded additively, following the GWAS conventions (Hill et al. 2008; Hivert et al. 2021). In other words, we assume the major allele is the reference (baseline) allele, and the minor allele is the alternative (effect) allele, thus, $P(G=0)={\left(1-p\right)}^{2},\;P(G=1)=2p(1-p)$, and $P(G=2)=p^{2}$.


$$\eta =X\beta :=1\beta_{0}+G\beta_{G}+E\beta_{E},$$

### 2.2 The Wald test

To test the association between SNP *G* and trait *Y*, i.e. $H_{0}:\beta_{G}=0$ versus $H_{1}:\beta_{G}\neq 0$, one can consider different tests such as the LRT, Score, or Wald tests. These tests have similar asymptotic behaviors under the null hypothesis, and they are locally equivalent (Rao et al. 1973; Serfling 2009). However, as noted by Demidenko (2007), these three likelihood-based tests differ globally. Since Wald tests are routinely used in GWAS, following the argument of Demidenko (2007), we carry out our power and sample size computation based on a Wald test.

The Wald test statistic in our setting is expressed as where $I_{X}^{-1}{\left(\hat{\beta }\right)}_{\left[2,2\right]}$ denotes the second diagonal element of the matrix $I_{X}^{-1}(\hat{\beta })$, and $\hat{\beta }$ is the maximum likelihood estimate (MLE) of β. $I_{X}(\beta )$ is the observed or conditional Fisher information matrix, conditional on the observed ***X***, defined as where W(β) is a *n *×* n* diagonal matrix, with the *i*th element as


$$T=\frac{{\hat{\beta }}_{G}{}^{2}}{I_{X}^{-1}{\left(\hat{\beta }\right)}_{\left[2,2\right]}},$$

$$I_{X}(\beta )=X^{T}W(\beta )X,$$

$$w_{i}={\left(\frac{\partial u_{i}}{\partial \eta_{i}}\right)}^{2}/\text{Var}(Y_{i}|X_{i}).$$

Under the null hypothesis, *T* is asymptotically $\chi_{1}^{2}$ distributed.

Using the above expression of *w_i_*, it is easy to see that when analyzing a continuous trait with residual variance $\sigma^{2}$ via linear regression (i.e. using the identity link function), $\partial u_{i}/\partial \eta_{i}=1$ and $w_{i}=1/\sigma^{2}$, which means $I_{X}(\beta )$ depends on $\sigma^{2}$ but not on any regression coefficients explicitly. In contrast, when analyzing a binary trait using logistic regression, which is a function of both *β_G_* and *β_E_*. Thus, the size of the nongenetic covariate effect *β_E_* explicitly influences the Fisher information matrix, hence the power analysis of *β_G_*.


$$w_{i}=\frac{\;\text{exp}(-\eta_{i})}{{\left(1+\text{exp}(-\eta_{i})\right)}^{2}},$$

### 2.3 The hidden factor in power and sample size computation

Assume the significance level of the test is *α*, and the sample size is large enough so that the asymptotic distribution of the Wald test statistic can be used. Let be the variance of ${\hat{\beta }}_{G}$, the power of the Wald test can be computed as where $Z_{1-\alpha /2}$ denotes the 1−α/2 quantile of the standard normal distribution. Worth reemphasizing is the fact that $V_{G,X}$, thus the power of logistic regression, explicitly depends on both *β_G_* and *β_E_*, as discussed in Section 2.2.


$$V_{G,X}:=I_{X}^{-1}{\left(\beta \right)}_{\left[2,2\right]}$$

$$\Phi \left(-Z_{1-\alpha /2}+\frac{\beta_{G}}{\sqrt{V_{G,X}}}\right)+\Phi \left(-Z_{1-\alpha /2}-\frac{\beta_{G}}{\sqrt{V_{G,X}}}\right),$$

The above power computation is for *conditional* power, conditional on the observed ***X***. However, for sample size determination for a successful replication study, the corresponding power analysis is performed prior to observing any data. In that case, the power is referred to as the *unconditional* power (Lyles et al. 2007).

To compute the unconditional power, naturally we replace the conditional Fisher information matrix $I_{X}(\beta )$ above with its unconditional version $I_{n}(\beta )$. Let $I_{x}(\beta )$ be the conditional Fisher information for a single observation x=(1,G,E) and $I_{1}(\beta )$ be its unconditional version. For the logistic regression considered, where the expectation is taken over, $F_{x}$, the distribution of the covariate space ***x*** (i.e. both *G* and *E*).


(2) $$\begin{array}{c} I_{1}(\beta )=E_{F_{x}}[I_{x}(\beta )]\\ =E_{F_{x}}[wx^{T}x]\\ =E_{F_{x}}[\frac{\;\text{exp}(-(\beta_{0}+\beta_{G}G+\beta_{E}E)}{{\left(1+\text{exp}(-(\beta_{0}+\beta_{G}G+\beta_{E}E))\right)}^{2}}\left[\begin{array}{ccc} 1 & G & E\\ G & G^{2} & GE\\ E & GE & E^{2} \end{array}\right]], \end{array}$$

Once $I_{x}(\beta )$ has been computed for a given $F_{x}$, the unconditional Fisher information matrix for a random sample of size *n* is


$$I_{n}(\beta ):=nI_{1}(\beta ).$$

The unconditional power is then where is based on the unconditional Fisher information matrix, $I_{n}(\beta )$.


(3) $$\Phi \left(-Z_{1-\alpha /2}+\frac{\beta_{G}}{\sqrt{V_{G,n}}}\right)+\Phi \left(-Z_{1-\alpha /2}-\frac{\beta_{G}}{\sqrt{V_{G,n}}}\right),\;$$

$$V_{G,n}:=I_{n}^{-1}{\left(\beta \right)}_{\left[2,2\right]}$$

To plan a successful replication study at the *α* level, the sample size *n* required to achieve a desirable power can be computed by simply inverting the power function, which is monotonic with respective to *n*. Although the sample size computation is for a specific genetic effect *β_G_*, it is clear that, similar to the conditional Fisher information, the unconditional Fisher information in Equation (2), therefore $V_{G,n}$ in Equation (3), also depends *β_E_*. Thus, this hidden factor must be explicitly accounted for when performing sample size calculation for a binary trait.

## 3 Methods

### 3.1 Designing the covariate space

To compute the unconditional Fisher information matrix $I_{n}(\beta )$, one needs to compute the moments and covariance of a random sample pair (*G_i_*, *E_i_*) from the corresponding covariate space $F_{x}$. An appropriately designed covariate space $F_{x}$ should be flexible enough to accommodate potential complex dependence structure between *G* and *E*, while conceptually simple enough so that that practitioners can make use of their domain knowledge.

In the work of Gauderman (2002b), the author implicitly assumed independence between *G* and *E*, requiring only the marginal distributions of *G* and *E*. Although this makes the method easy-to-implement, the assumption may not hold in practice (Plomin et al. 1977; Scarr and McCartney 1983; Knafo and Jaffee 2013; Zhu et al. 2018; Namjou et al. 2019). Furthermore, the implemented software Quanto only allows users to specify *E* when the target analysis is the *G *×* E* interaction effect, not the main effect of *G*.

The work of Demidenko (2007), on the other hands, allows $F_{x}$ to accommodate dependence between a binary *G* and a binary *E*, by introducing a second-stage logistic regression, where $\gamma_{0}^{*}$ is determined by user-specified marginal probabilities of *G* and *E*. As a result, users only need to additionally input the knowledge about $\gamma_{E}^{*}$ to fully specify $F_{x}$. However, the method of Demidenko (2007) is designed for a binary *G* (hence the typical GWAS additive coding of *G* not applicable) and a binary *E*, and its generalization to different types of *G* and *E* is nontrivial.


(4) $$\log\left(\frac{P(G=1|E)}{P(G=0|E)}\right)=\gamma_{0}^{*}+\gamma_{E}^{*}E,\;$$

Here, we utilize the idea of second-stage regression (Demidenko 2007) but extend it to a more general setting. Instead of treating *E* as a covariate in the second-stage regression, we consider it to be the response variable such that, where *g*_2_ is the link function, being identity when *E* is continuous and logit when *E* is binary.


(5) $$g_{2}(E(E|G))=\gamma_{0}+\gamma_{G}G,\;$$

Compared with the second-stage regression model in Equation (4), the proposed method can accommodate different types of *E* in a unified framework. When *E* is continuous, the regression model also requires Var(E|G) in order to be fully specified. The value of Var(E|G) can be computed based on user-provided information such as $\mu_{E},\sigma_{E}$, and *p*.

Since the intercept parameters (for both Stages 1 and 2) do not have clear interpretations in logistic regression, particularly for case–control studies (Castelloe 2018), the default implementation in our software only requires users to input the nonintercept regression coefficients, and the marginal information (e.g. disease prevalence). All the intercept parameters are then automatically gleaned by the software.

We note that the proposed method can account for multiple *E*s by specifying the corresponding *g*_2_ function for each *E* considered. Later, we will demonstrate the utility of our approach in a UK Biobank data application of hypertension, for which both age and sex are important covariates to consider for power and sample size computation. In the rest of this section, we assume there is only one covariate *E* to simplify the presentation.

### 3.2 Proposed method 1: semi-simulation (P1.SS)

The estimation of the unconditional power heavily depends on the computation of $I_{n}(\beta ):=nI_{1}(\beta )$. Unfortunately, unless in some special cases such as when both *G* and *E* are binary, $I_{1}(\beta )$ in Equation (2) does not have a closed-form expression for a general $F_{x}$ (Demidenko 2007). Thus, to estimate $I_{n}(\beta )$, we propose to use a sample estimate.

Specifically, for a large integer *B*, we simulate independent observations ${\left\{G_{i},E_{i}\right\}}_{i=1}^{B}$ from the covariate space $F_{x}$, and for each $x_{i}=(1,G_{i},E_{i})$ we compute the corresponding conditional Fisher information matrix, where


$$I_{x_{i}}(\beta )=x_{i}{}^{T}w_{i}(\beta )x_{i},$$

$$w_{i}(\beta )=\frac{\;\text{exp}(-(\beta_{0}+\beta_{G}G_{i}+\beta_{E}E_{i})}{{\left(1+\text{exp}(-(\beta_{0}+\beta_{G}G_{i}+\beta_{E}E_{i}))\right)}^{2}}.$$

By a simple application of the law of large number, the sample estimate, converges almost surely to the true expected matrix $I_{n}(\beta )$ as *B* grows.


(6) $${\tilde{I}}_{n}(\beta ):=\frac{\sum_{i=1}^{B}nI_{x_{i}}(\beta )}{B},\;$$

As we will illustrate later in the simulation studies, ${\tilde{I}}_{n}(\beta )$ exhibits little variation for large *B* (e.g. >10 000). Furthermore, the proposed *semi-simulation* method is scalable, as for each $I_{x_{i}}(\beta )$ we only compute the observed Fisher information matrix for one single observation. Thus, the computational load depends on *B* but is independent of the target sample size *n*. Once $I_{n}(\beta )$ is replaced by ${\tilde{I}}_{n}(\beta )$, the power computation can proceed using Equation (3), and sample size estimation by inverting the power function.

This semi-simulation approach is computationally more efficient than the traditional full-simulation approach. Given a sample size *n*, a full-simulation approach requires simulating *B* independent datasets, ${\left\{{\left(G_{i}^{b},E_{i}^{b},Y_{i}^{b}\right)}_{i=1}^{n}\right\}}_{b=1}^{B}$, computing the test statistic for each dataset *b*, and then calculating the empirical power which is the proportion of significant tests among all simulated datasets (Kumle et al. 2021). In contrast, the proposed semi-simulation approach simulates only one dataset that consists of covariates ${\left(G_{i},E_{i}\right)}_{i=1}^{B}$, where *B* is a large number that does not depend on the sample size *n*, and it avoids the repeated computations of the test statistics. Since the computation of the proposed semi-simulation method does not depend on the sample size, it is also more scalable than the full-simulation approach, e.g. to biobank-sized data. Furthermore, using the semi-simulation method, the replication sample size for a desirable power can be efficiently computed by inverting the closed-form power function in Equation (3).

### 3.3 Proposed method 2: representative dataset (P2.RD)

An alternative method that does not rely on plugging in the sample estimate of $I_{n}(\beta )$ is through the use of a *representative* dataset, an idea that was originally suggested by O’Brien (1986) and later extended by Lyles et al. (2007). This idea using a representative dataset for power computation is not new. For example, it has been implemented in the commercial SAS software, with its CUSTOM option, as discussed in Castelloe (2018).

In our setting, given a sample size *n*, assume there exists a representative covariate sample ${\left\{x_{i}\right\}}_{i=1}^{n}={\left\{(1,G_{i},E_{i})\right\}}_{i=1}^{n}$ from the covariate space $F_{x}$, which we define later. We then expand ${\left\{x_{i}\right\}}_{i=1}^{n}$ to consider both possible outcomes of the binary trait, so that each observation $x_{i}$ splits into $\left\{x_{i},y_{i}=0\right\}$ and $\left\{x_{i},y_{i}=1\right\}$. Additionally, each expanded observation is given a weight, so that $\delta_{i}^{0}+\delta_{i}^{1}=1$, where


(7) $$\delta_{i}^{l}=P(y_{i}=l|x_{i})=\frac{\;\text{exp}\;{\left(\beta_{0}+\beta_{G}G_{i}+\beta_{E}E_{i}\right)}^{l}}{1+\text{exp}(\beta_{0}+\beta_{G}G_{i}+\beta_{E}E_{i})},$$

For *l* = 0 and 1.

Thus, the original representative dataset ${\left\{x_{i}\right\}}_{i=1}^{n}$ is now expanded into the following representative dataset, which has 2*n* (weighted) observations. Standard MLE of $V_{G,n}$, derived from the corresponding weighted log-likelihood, yields ${\hat{V}}_{G,n}$, which can be directly plugged into Equation (3) to complete the power computation (Lyles et al. 2007).


(8) $$\mathrm{RD}:={\left\{\begin{array}{ccc} x_{i}, & y_{i}=0, & \delta_{i}^{0}\\ x_{i}, & y_{i}=1, & \delta_{i}^{1} \end{array}\right\}}_{i=1}^{n},\;$$

It remains to be discussed what is a representative ${\left\{x_{i}\right\}}_{i=1}^{n}$ and how the expanded representative dataset RD can be obtained in our study setting. In the case of conditional power analysis where covariates are already observed, the observed ${\left\{x_{i}\right\}}_{i=1}^{n}$ can be directly used in Equation (7) to establish the representative dataset of Equation (8).

For the unconditional power analysis, ${\left\{x_{i}\right\}}_{i=1}^{n}$ can be obtained by using user-provided $F_{x}$. Lyles et al. (2007) provided examples on how to define the notion of representative dataset for different types of $F_{x}$. We follow the procedures of Lyles et al. (2007) for the types of $F_{x}$ considered in Section 3.1.

When *E* is binary and the link function in Equation (5) is logistic, we can compute the expected counts for category {(G=i,E=j)}, *i *=* *0, 1, and 2, and *j *=* *0 and 1, as using the available information such as MAF and the inheritance mode, with appropriate rounding to ensure that $n_{i,j}$s are integers and sum to *n*.


$$n_{i,j}=nP(G=i,E=j),$$

When *E* is continuous and the link function is identity with $\text{Var}(E|G)=\sigma_{E}^{2}$, we first categorize the dataset based on *G* such that $n_{i}=nP(G=i)$, *i *=* *0, 1, and 2. Then for each of the $j=1,\ldots ,n_{i}$ observations of ${\left\{G_{j}=i\right\}}_{j=1}^{n_{i}}$, where $\Phi^{-1}$ is the inverse of the cumulative distribution function of the standard normal.


$$E_{j}=\gamma_{0}+\gamma_{G}i+\sigma_{E}\Phi^{-1}[(j-0.375)/(n_{i}+0.25)],$$

Although Castelloe (2018) has provided a general guideline on how to implement the representative dataset (RD) approach, the commercial SAS software requires users to manually create an RD by themselves, which can be challenging without significant coding experience. In comparison, our open-source software is more accessible as it automatically creates RD based on user-specified parameter values.

## 4 Simulation studies

### 4.1 Overview of the simulation design

We compared the power and sample size computed using the proposed P1.SS and P2.RD methods (implemented as the R package SPCompute available at CRAN) with those computed using Quanto of Gauderman (2002b) (version 1.2.4), and the method of Demidenko (2007) using its web-platform (dartmouth.edu/∼eugened/power-samplesize.php).

We considered three different scenarios for $F_{X}$, including no covariate *E* (as Quanto does not allow for *E*), *E* being binary [as the method of Demidenko (2007) only allows for binary *E*], and *E* being continuous. Although we only considered one *E* in the simulation studies for method comparison, our implemented SPCompute R package allows for multiple *E*s, as demonstrated in our UK Biobank application study in Section 5. Finally, for the simulation studies we also considered three study designs, where S1 is case–control retrospective (Quanto only allows for the case–control study design), while S2 and S3 are prospective to reflect the design of the emerging biobank-sized data such as the UK Biobank data used in our application.

In the power computation, the log odds ratio (OR) for the genetic effect was fixed at $\beta_{G}=\text{log}(1.5)$, across a range of covariate effects, in the simulation settings S1 and S2. As S3 considers continuous covariates, to make the power range comparable with S1 and S2, the log OR (*β_G_*) was fixed at log(1.3) in S3. For completeness, we also considered a range of OR values, and for each we computed the necessary replication sample size to achieve 80% power. By convention, the same significance level 0.05 was used for both the power and replication sample size computation.

The accuracy of each method was then assessed by comparing the computed power with the empirical value, obtained through a large number of independent replications for a range of sample sizes (600–10 000). First, for each given sample size, we obtained the computed power for each method considered. Second, we simulated 1000 independent replications to obtain the empirical power, which was then used as the oracle value to benchmark and obtain the absolute error (AE). Finally, we summarized the AE values with their averages and maximums across the range of sample sizes considered (Table 1).

**Table 1:** The average and maximum AE, across different sample sizes, between the oracle and computed power using the different methods for the three scenarios considered.^a^

	Scenario 1 (S1)		Scenario 2 (S2)		Scenario 3 (S3)	
Methods	Average AE	Maximum AE	Average AE	Maximum AE	Average AE	Maximum AE
P1.SS	0.008	0.022	0.010	0.021	0.015	0.032
P2.RD	0.009	0.019	0.012	0.023	0.012	0.027
Demidenko	0.009	0.019	0.012	0.024	0.124	0.194
					0.097*	0.150*
Quanto	0.009	0.022	0.052	0.084	0.112	0.173

a P1.SS is the proposed “semi-simulation” method in Section 3.2, and P2.RD is the proposed “representative dataset” method in Section 3.3. In Scenario 3, the method of Demidenko (2007) was implemented by dichotomizing *E* or without considering *E* (results*). See legend to Supplementary Fig. S1 for additional details.

#### 4.1.1 Scenario 1: no covariate E with a case–control retrospective study design

The choice of no covariate effect was to accommodate the implementation of Quanto of Gauderman (2002b). Without loss of generality, the disease prevalence was assumed to be 20%, and the observations were obtained independently with a retrospective sampling design and the standard case-to-control ratio of 1-to-1.

The associated SNP has a MAF of 0.1, with a dominant effect *β_G_* ranging from log(1.1) to log(2.5) in the replication sample size computation and log(1.5) in the power computation. The choice of a dominant genetic effect was to accommodate the implementation of Demidenko (2007) method, which only allows for a binary *G*. Finally, we used $\beta_{0}=-2$, though we note that the intercept parameter does not affect the power of a case–control study.

#### 4.1.2 Scenario 2: binary E with a prospective study design

Similar to S1 above, in the second scenario the disease prevalence is also 20%, and the associated SNP with MAF of 0.1 has a dominant effect *β_G_* ranging from log(1.1) to log(2.5) in the replication sample size computation and log(1.5) in the power computation. However, the observations were obtained independently with a prospective sampling design as in the UK Biobank data. Additionally, the nongenetic covariate *E* has a population exposure rate of P(E=1)=0.3 with effect $\beta_{E}=\text{log}(2.5)$. Finally, $\gamma_{G}=\text{log}(0.2)$, quantifying the dependency between *G* and *E* as defined in Equation (5).

To implement Quanto of Gauderman (2002b), which only allows for case–control study design, we used a case-to-control ratio of 1-to-4 to approximate the result for a disease with prevalence of 20%. Additionally, when the power analysis is about *G* main effect (as opposed to *G *×* E* interaction effect), Quanto does not consider the presence of *E*. Thus, we only input the information about *G* in the implementation of Quanto. The method of Demidenko (2007) accommodates the presence of one binary covariate *E* for the power (and sample size) computation; *G* must be binary, hence the dominant genetic model was assumed for method implementation.

#### 4.1.3 Scenario 3: continuous E with a prospective study design

For this last scenario, without loss of generality, the covariate *E* was assumed to follow the standard normal distribution conditional on *G*. The dependency between *G* and *E* was set to $\gamma_{G}=\text{log}(0.5)$. All other model specifications are the same as in S2 above, including the disease prevalence (20%), MAF (0.1), the genetic effect [ranging from log(1.1) to log(2.5) in the replication sample size computation and log(1.3) in the power computation], and the nongenetic covariate effect log(2.5).

As in the previous scenario, we ignored the information on *E* for the implementation of Quanto. For the method of Demidenko (2007), which only allows for a binary *E*, we considered two approaches. We first omitted the continuous covariate *E* (corresponding results*), and we then dichotomized *E* by defining $\tilde{E}:=I(E>0)$. This corresponds to creating two mis-specified models, and


(9) $$\log\left(\frac{P(Y=1|G,\tilde{E})}{P(Y=0|G,\tilde{E})}\right)=\beta_{0}+\beta_{G}G+{\tilde{\beta }}_{E}\tilde{E},\;$$

(10) $$\log\left(\frac{P(\tilde{E}=1|G)}{P(\tilde{E}=0|G)}\right)={\tilde{\gamma }}_{0}+{\tilde{\gamma }}_{G}G.\;$$

As the parameter values specified for the true models (1) and (5) cannot be directly used for the two mis-specified models, we used estimated ${\tilde{\gamma }}_{G}$ and ${\tilde{\beta }}_{E}$. We first simulated a large a number of observations ${\left\{G_{i},E_{i},Y_{i}\right\}}_{i}^{3\times 10^{5}}$ using the true model. We then dichotomized the continuous *E* to obtain $\tilde{E}$ as specified above. Finally, we regressed *Y* on *G* and $\tilde{E}$, and $\tilde{E}$ on *G* to obtain sample estimates of ${\tilde{\beta }}_{E}$ and ${\tilde{\gamma }}_{G}$ for the second implementation of the method of Demidenko (2007).

#### 4.1.4 Methods comparison across the three scenarios

Table 1 and Supplementary Fig. S1 show the superior performances of the proposed two methods, as P1.SS and P2.RD have smaller average and maximum AE compared with the true power. The empirical results are consistent with our analytical expectation: Ignoring covariate effect, *at the (replication) study planning stage*, can lead to overestimated power and hence underestimated replication sample size for studying a binary trait.

In the presence of a binary covariate (S2), Quanto tends to *overestimate* the power of an association study, while both Demidenko and the proposed two methods provide power estimates close to the Oracle values. However, if the influential covariate is continuous (S3, e.g. age as in our UK Biobank application for hypertension), only the proposed two methods (P1.SS and P2.DD) perform well.

In the right panel of Supplementary Fig. S1b, d, and f shows the estimated sample sizes, necessary to achieve 80% power at the 0.05 level, to successfully replicate an associated SNP with OR ranges from 1.1 to 2.5. Similar conclusion can be drawn here, as the existing methods tend to *underestimate* the necessary sample size for a successful replication study in the presence of influential covariate, while the proposed P1.SS and P2.RD methods are accurate.

To examine the sensitivity of the proposed methods to the choice of MAF, we also computed the average and maximum AE of the two proposed methods under the same setting in Scenario 3 (S3) above, for a range of MAF from 0.05 to 0.5. As shown in Supplementary Fig. S2, the average and maximum AE values are both stable across MAF, illustrating the robustness of the proposed methods.

### 4.2 Choice between the proposed P1.SS and P2.RD methods from the computational perspective

To select between the two proposed methods, P1.SS and P2.RD as, respectively, described in Sections 3.2 and 3.3, here we study factors influencing the computational efficiencies of the two methods and make recommendations to practitioners.

Conceptually, the computational efficiency of the semi-simulation P1.SS method depends on *B*, the number of independent observations drawn from $F_{X}$ in order to obtain ${\tilde{I}}_{n}(\beta )$ in Equation (6). As ${\tilde{I}}_{n}(\beta )$ is based on *B* replicates of one-sample $I_{x_{i}}(\beta )$, the targeted sample size *n* does not have a direct impact on computational time. In contrast, the computational time of the P2.RD method depends on *n*, as the method first creates a representative dataset of size *n* from $F_{X}$ then expand it to weighted 2*n* observations as in Equation (8).

**Figure 1:**
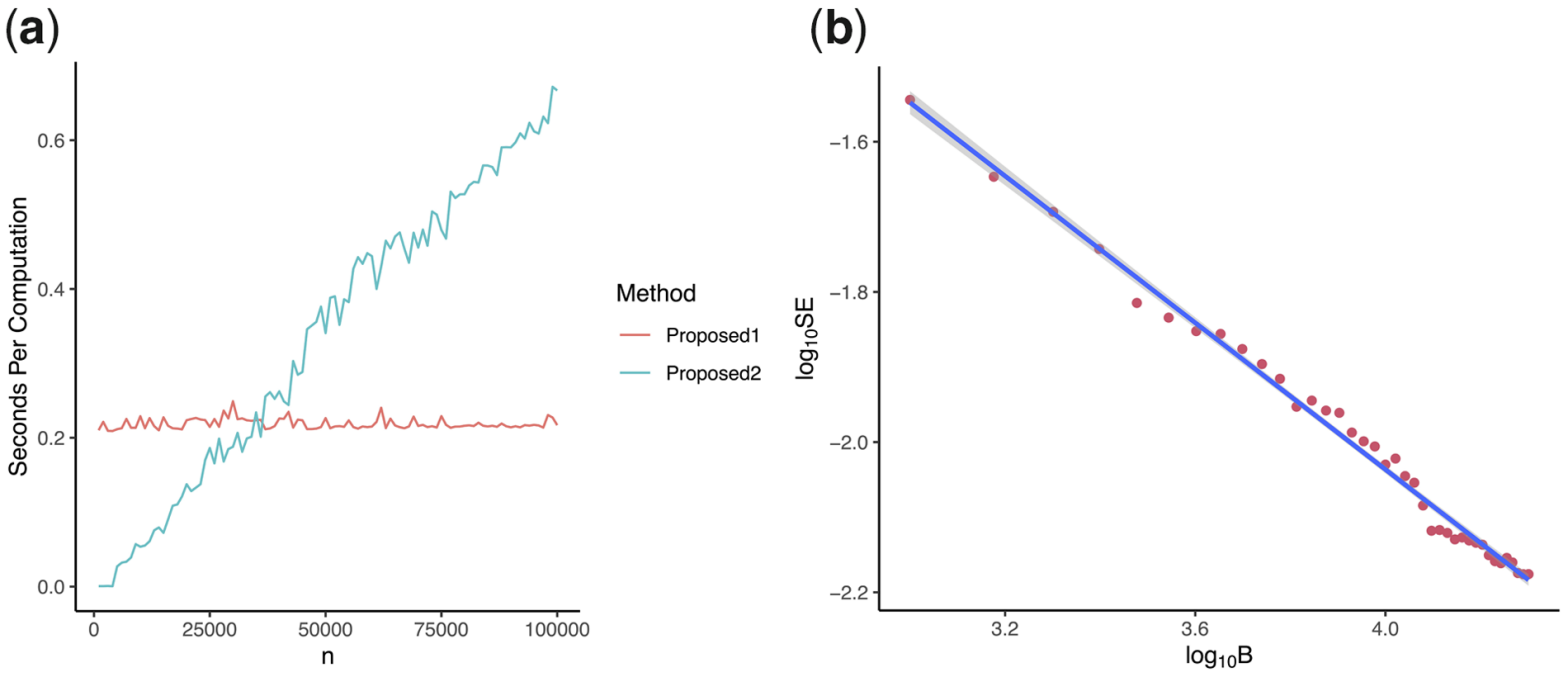
(a) The relationship between the run time in seconds per computation for each method across different sample sizes, using simulation Scenario 2; results for the other two scenarios are characteristically similar. The Proposed 1 is the “semi-simulation” method (P1.SS) in Section 3.2, The proposed 2 is the “representative dataset” method (P2.RD) in Section 3.3. (b) There is a linear relationship between the ${\;\text{log}}_{10}$ SE of estimated power and the ${\;\text{log}}_{10}$ number of replicates (B) used for the proposed “semi-simulation” method 1

To numerically demonstrate the computational properties of the two methods, without loss of generality, we considered simulation Scenario S2 in Section 4.1.2 and used $\beta_{G}=\text{log}(1.5)$ for illustration. Results in Fig. 1a confirm our analytical expectation: The computational time of P2.RD grows linearly with respect to *n*, while that of P1.SS is independent of *n*.

However, the accuracy of P1.SS depends on large *B*; we used B= 10 000 (${\;\text{log}}_{10}(B)=4$) in Fig. 1a. Figure 1b shows the stability of P1.SS with respect to ${\;\text{log}}_{10}(B)$. For each choice of ${\;\text{log}}_{10}(B)$ from 3.0 to 4.3, the ${\;\text{log}}_{10}$ sample standard error of the power (SE) computed by P1.SS, obtained from 1000 independently simulated replicates, was shown in Fig. 1b. Results clearly show that B≥ 10 000 (${\;\text{log}}_{10}(B)\geq 4$) leads to negligible SE of <0.01 (${\;\text{log}}_{10}(\text{SE})<-2$). Thus, the default value for *B* in our method implementation is 10 000. Interestingly, the relationship between *B* and SE appears to be approximately log-log linear.

We note that *Y*-axis in Fig. 1a measures the run time in seconds for computation of one set of parameter values (i.e. per computation). In practice, it is often necessary to run power and sample size analysis for a fine grid of a large number of possible parameter values. Thus, the run time difference aggregates and can differ significantly between P1.SS and P2.RD. In general, when *n* is larger than 25 000, P1.SS is preferred over P2.RD. Although both the RD and SS methods are included in our software SPCompute, the RD approach is not scalable. Thus, RD is only suggested for power computation at medium sample size, and SS is set as the default approach in our software.

## 5 Application to the UK Biobank data

To illustrate the practical utility of the proposed power and sample size computation methods, we applied them to the UK Biobank data (Sudlow et al. 2015; Bycroft et al. 2018), focusing on the understudied participants with recent African ancestry, but also the European sample as another illustration; see Section 5.4. Without loss of generality, we chose hypertension as the binary trait of interest. For completeness, we also analyzed (diastolic) blood pressure, a continuous trait to contrast with the binary trait.

### 5.1 The African sample and SNP data quality control

We started with the 3460 participants with self-reported ancestry being African. First, we followed the standard practice (Marees et al. 2018) to filter out individuals with genotype missingness higher than 20%. To remove related individuals, we then filtered out individuals with kinship coefficient larger than 0.25, which ended up with 3182 unrelated or distantly related self-reported Africans.

To account for heterogeneity in self reporting, we then performed principle component analysis (PCA) using the overall principle components (PCs) provided by UKB (Data-Field 22009). Supplementary Figure S2a shows the first two PCs of all UKB samples, stratified by self-reported Africans and Others, which suggests heterogeneity in self reporting. We then applied a *K*-mean algorithm with *K *=* *4 (Hartigan and Wong 1979) to the 3182 unrelated self-reported Africans (Supplementary Fig. S2b–d) using the overall PCs provided. Cluster 1 contains 2601 individuals, 75% of all self-reported African participants (Supplementary Fig. S2b–d). Following the common practice, we also computed new PCs using only the 3182 individuals (Supplementary Fig. S3). Among the 2601 individuals in Cluster 1, we then removed 91 individuals whose new PCs were 4 SD away from the mean. Thus, the final GWAS sample consists of n= 2510 unrelated individuals with PC-defined ancestry.

For the genetic data, we started with 784 256 genotyped autosomal SNPs (Data-Field 22418), and we then filtered out SNPs based on the thresholds of HWE *P*-value <1e−10, MAF < 0.01, and missing rate >0.2. The X chromosome was not included in our analysis due to the recent report of previously unrecognized data quality issue of the X chromosome (Wang et al. 2022). In total, 379 003 common, good quality autosomal SNPs were selected for the subsequent analyses.

### 5.2 GWAS of hypertension and blood pressure

We considered two phenotypes, one binary (hypertension) and the other continuous (diastolic blood pressure). In this application, we only considered their measurements at the initial assessment, as longitudinal data analysis is beyond the scope of this work. Additionally, for blood pressure we considered the automated reading (Data-Field 4079) instead of the manual reading. There are two automated measurements of blood pressure during the initial assessment, and we used the average. Among the *n* = 2510 analyzed individuals, the prevalence rate of hypertension (Data-Field 20002, Data-Coding 1065) is 39.48%, and the diastolic blood pressure has mean 85.35 and SD 10.75.

The GWAS of both traits included age and sex as covariates, and are carried out using the software PLINK 2.0 (Purcell et al. 2007). Of the 2510 individuals, 1232 are females, and 1278 males, and the mean (SD) age is 51.2 (7.9) years. The analyses of the binary hypertension trait and the continuous blood pressure outcome used logistic and linear regression, respectively. The two GWAS results are displayed in Supplementary Fig. S4a and b, respectively, for hypertension and blood pressure. Given the small sample size, it is not surprising that none of the SNPs reached the genome-wide significance of 5e−8 (Dudbridge and Gusnanto 2008).

### 5.3 Power and sample size computation for the African sample

To illustrate the importance of including covariates in power and sample size computation for a binary trait, as a proof-of-principle, we focused on the top five-ranked SNPs from each GWAS. The effect size estimates of both SNPs and covariates were used for the corresponding power and sample size computation, though we recognize the potential issue of winner’s curse (Sun et al. 2011); this issue does not change characteristically the conclusion drawn from the methods comparison. The power computation in this section was carried out through the recommended P1.SS method, which is the default option in our software. Thus, the computational efficiency of the power computation does not depend on the sample size.

To show the important role of age and sex in study planning of the binary trait of hypertension, we computed powers and required replication sample sizes twice. We first ignore the two covariates as commonly done (without E), which is equivalent to using Quanto; the method of Demidenko (2007) is not applicable as there are two covariates. We then accounted for covariate effects (with E) using the proposed method P1.SS, the default method implemented in SPcompute. Finally, in addition to α=0.05, the standard significance level used for planning a successful replication study, we also considered 5e−8, the genome-wide significance level to demonstrate the power and sample size needed for the *discovery* GWAS to achieve 80% power. For comparison, we also analyzed the continuous trait of blood pressure, where the covariate effects are implicitly incorporated through the specification of the residual variance.

**Figure 2:**
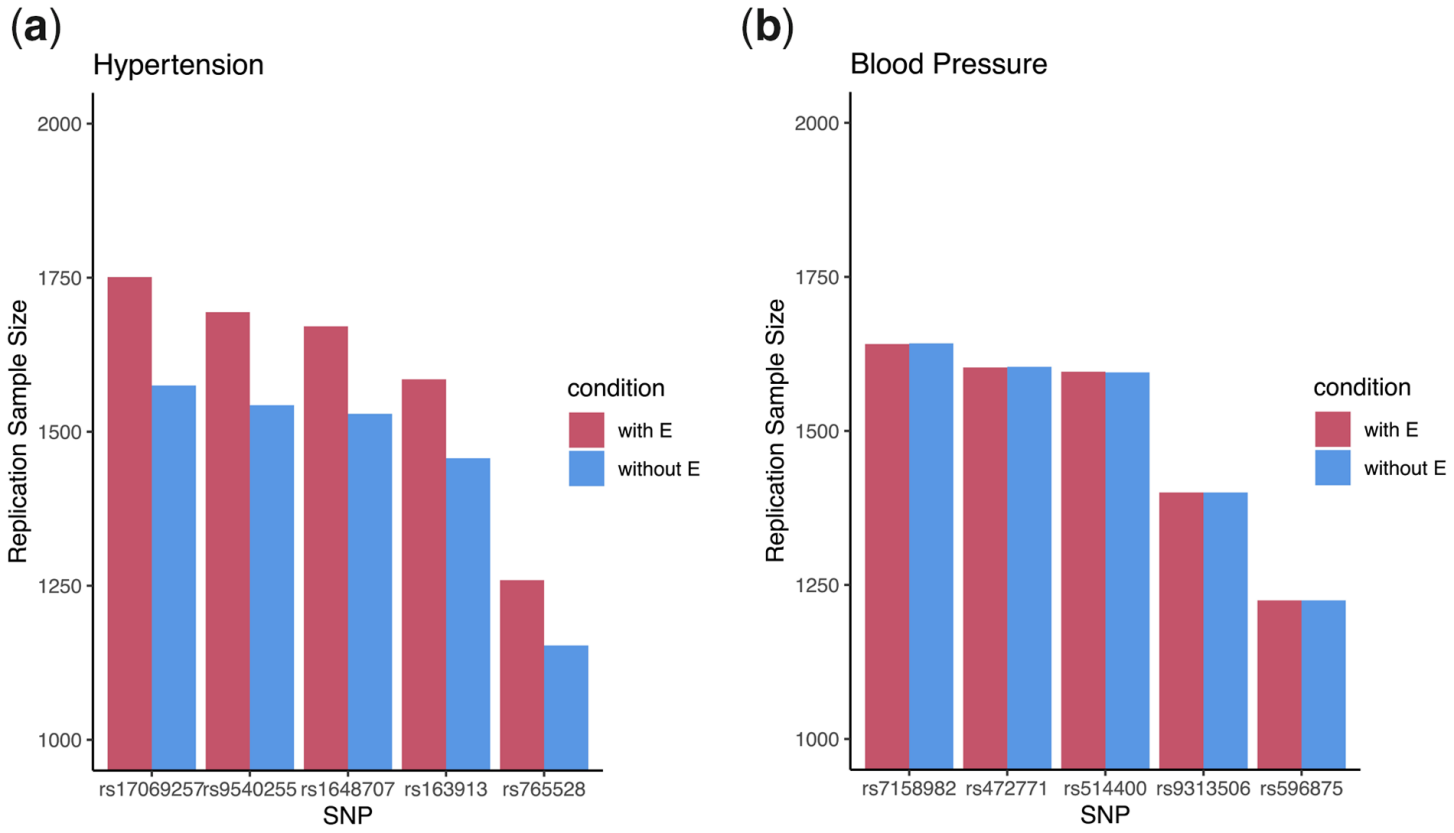
Sample sizes estimation for study planning of the top five-ranked SNPs identified in GWAS of the binary hypertension trait (a) and the continuous diastolic blood pressure trait (b), using the African sample (*n* = 2510) identified through a PCA analysis of the self-identified African sample of the UK Biobank data as discussed in Section 5. The genetic effects of these SNPs used for power and sample size computations are based on a standard GWAS where age and sex were included as important covariates. For (replication) study planning, the red bars are the computed power or sample size with adjustment for age and sex, and the blues bars are the values without explicitly considering age and sex. The two approaches do not have difference in power and sample size planning for the continuous blood pressure trait, as age and sex effects are incorporated through residual variance. In contrast, when analyzing a binary trait, the higher blue bars in (a) show that ignoring covariate effects leads to *underestimated replication sample size* (for 80% power at *α* = 0.05)

For each trait analyzed, the computed replication sample sizes for the top SNPs are shown in Fig. 2, which are consistent with our simulation results. For the binary hypertension trait (the left panel of Fig. 2), the higher blue bars in Fig. 2a show that ignoring covariate effects leads to *underestimated replication sample size* (for 80% power at α= 0.05) when planning a replication study of a binary trait. In contrast, when studying the continuous blood pressure trait, age, and sex effects are already implicitly incorporated through the residual variance. Thus, covariate effects *β_E_*s do not have to be explicitly included in the power and sample size computation for a continuous trait.

### 5.4 The European sample

For completeness, we carried out the GWAS of hypertension and blood pressure again using the European sample selected based on their genetic PCs (Field ID: 22006, *n *=* *276 682; Supplementary Fig. S6), and then performed similar power (and sample size) computations. In this analysis, we focused on the five genome-wide-significant SNPs with the least significance; the most significant ones are uninteresting with power close to 1. The conclusion is similar to what we observed in the analysis of the African sample, where the omitted covariates (i.e. age and sex) severely affected the analysis of the binary trait of hypertension but not the continuous blood pressure (Supplementary Fig. S8). Supplementary Table S2 shows the GWAS summary statistics of these five SNPs. As expected, the estimated genetic effect sizes of these SNPs are substantially smaller than those selected in the African sample (Supplementary Table S1), which explains the larger replication sample size for the SNPs in the European sample.

## 6 Discussion

Explicitly adjusting for covariate effects is a standard practice in GWAS, but it is rarely done at the study planning stage. When performing power estimation or replication sample size calculation for a continuous trait through linear regression, covariate effects are implicitly accounted for through residual variance. However, when analyzing a binary trait through logistic regression, covariate effects (*β_E_*s) must be explicitly specified and included in power and sample size computation, in addition to the genetic effect of interest (*β_G_*).

This phenomenon is closely related to the noncollapsibility of the logistic regression (Gail et al. 1984) in the statistics literature, but tools available to practitioners are limited. In this work, we developed and implemented a flexible software SPCompute for accurate and efficient power and sample size computation for a binary trait. We applied the proposed method to the UK Biobank data, analyzing the binary hypertension trait and simultaneously accounting for age and sex covariate effects in power and sample size computation. We also conducted extensive simulation studies to demonstrate the accuracy and efficiency of the proposed method.

However, there are still several limitations of the proposed method that require future work to address. For example, winner’s curse where the effect size estimates of significant SNPs are biased upward is known to be a common problem in GWAS (Sun and Bull 2005; Zöllner and Pritchard 2007; Zhong and Prentice 2010; Sun et al. 2011). Therefore, it would be of interest to investigate how SPCompute accounts for the winner’s curse. Another direction of extension would be to account for mis-classification (particularly the control data), which affects the power of an association study (Rekaya et al. 2016; Lin et al. 2020; Zhang and Yi 2020). Additionally, the proposed framework can be further generalized to accommodate the simultaneous analysis of multiple rare variants (Derkach et al. 2014; Li et al. 2019, 2020, 2022). Finally, the proposed method assumes a random sample of unrelated individuals. Power and sample size computation for related individuals are worthy of future work.

Our UKB application in Section 5 only serves as a proof-of-principle and highlights the practical utility of SPCompute, such as its ability to handle both binary and continuous covariates. We made some simplifying assumptions to make the example easier to understand. For example, we accounted for the covariate effects of age and sex simultaneously by introducing two separate models in the second-stage regression of Equation (5). This implicitly assumed the two covariates are conditionally independent given the SNP *G*, an assumption that might be unrealistic in more complex settings. In general, the proposed methods can also handle multiple *E*s that are correlated conditional on *G*, but at the cost of user’s specifying nested second-stage GLMs; see user manual of *SPCompute v1.0.3* for additional details. Finally, the framework of the proposed method can be generalized to incorporate gene–gene and gene–environment interaction effects, which we will provide as future software updates.

## Supplementary Material

btad139_Supplementary_Data

## Data Availability

This research has been conducted using the UK Biobank Resource under Application Number 64875. Data are available at www.ukbiobank.ac.uk/ with the permission of UK Biobank. The simulated datasets can be found at the online repository of this article.

## References

[btad139-B1] Bycroft C , Freeman C, Petkova D et al The UK Biobank resource with deep phenotyping and genomic data. Nature 2018;562:203–9. 30305743 10.1038/s41586-018-0579-z PMC6786975

[btad139-B2] Castelloe J. Power analysis for generalized linear models using the new custom statement in proc power. In: *Proceedings of the SAS Global Forum 2018 Conference*. Cary, NC: SAS Institute Inc., 2018.

[btad139-B3] Demidenko E. Sample size determination for logistic regression revisited. Stat Med 2007;26:3385–97. 17149799 10.1002/sim.2771

[btad139-B4] Demidenko E. Sample size and optimal design for logistic regression with binary interaction. Stat Med 2008;27:36–46. 17634969 10.1002/sim.2980

[btad139-B5] Derkach A , Lawless JF, Sun L. Pooled association tests for rare genetic variants: a review and some new results. Stat Sci 2014;29:302–21.

[btad139-B6] Dudbridge F , Gusnanto A. Estimation of significance thresholds for Genomewide Association scans. Genet Epidemiol 2008;32:227–34. 18300295 10.1002/gepi.20297 PMC2573032

[btad139-B7] Gail MH , Wieand S, Piantadosi S. Biased estimates of treatment effect in randomized experiments with nonlinear regressions and omitted covariates. Biometrika 1984;71:431–44.

[btad139-B8] Gauderman WJ. Sample size requirements for association studies of gene-gene interaction. Am J Epidemiol2002a;155:478–84. 11867360 10.1093/aje/155.5.478

[btad139-B9] Gauderman WJ. Sample size requirements for matched case-control studies of gene–environment interaction. Stat Med2002b;21:35–50. 11782049 10.1002/sim.973

[btad139-B10] Golan D , Lander ES, Rosset S. Measuring missing heritability: inferring the contribution of common variants. Proc Natl Acad Sci USA 2014;111:E5272–E5281. 25422463 10.1073/pnas.1419064111 PMC4267399

[btad139-B11] Hartigan JA , Wong MA. Algorithm as 136: a k-means clustering algorithm. J R Stat Soc Ser C (Appl Stat) 1979;28:100–8.

[btad139-B12] Hill WG , Goddard ME, Visscher PM. Data and theory point to mainly additive genetic variance for complex traits. PLoS Genet 2008;4:e1000008. 18454194 10.1371/journal.pgen.1000008 PMC2265475

[btad139-B13] Hivert V , Sidorenko J, Rohart F et al Estimation of non-additive genetic variance in human complex traits from a large sample of unrelated individuals. Am J Hum Genet 2021;108:786–98. 33811805 10.1016/j.ajhg.2021.02.014 PMC8205999

[btad139-B14] Hong EP , Park JW. Sample size and statistical power calculation in genetic association studies. Genomics Inform 2012;10:117. 23105939 10.5808/GI.2012.10.2.117 PMC3480678

[btad139-B15] Hsieh F. Sample size tables for logistic regression. Stat Med 1989;8:795–802. 2772439 10.1002/sim.4780080704

[btad139-B16] Hsieh FY , Bloch DA, Larsen MD. A simple method of sample size calculation for linear and logistic regression. Stat Med 1998;17:1623–34. 9699234 10.1002/(sici)1097-0258(19980730)17:14<1623::aid-sim871>3.0.co;2-s

[btad139-B17] Mayhew AJ , Meyre D. Assessing the heritability of complex traits in humans: methodological challenges and opportunities. Curr Genomics 2017;18:332–40. 29081689 10.2174/1389202918666170307161450 PMC5635617

[btad139-B18] Knafo A , Jaffee SR. Gene–environment correlation in developmental psychopathology. Dev Psychopathol 2013;25:1–6. 23398748 10.1017/S0954579412000855

[btad139-B19] Korte A , Farlow A. The advantages and limitations of trait analysis with GWAS: a review. Plant Methods 2013;9:1–9. 23876160 10.1186/1746-4811-9-29 PMC3750305

[btad139-B20] Kumle L , Võ ML-H, Draschkow D. Estimating power in (generalized) linear mixed models: an open introduction and tutorial in R. Behav Res Methods 2021;53:2528–43. 33954914 10.3758/s13428-021-01546-0 PMC8613146

[btad139-B21] Li X , Li Z, Zhou H et al Dynamic incorporation of multiple in silico functional annotations empowers rare variant association analysis of large whole-genome sequencing studies at scale. Nat Genet 2020;52:969–83. 32839606 10.1038/s41588-020-0676-4 PMC7483769

[btad139-B22] Li Z , Li X, Liu Y et al Dynamic scan procedure for detecting rare-variant association regions in whole-genome sequencing studies. Am J Hum Genet 2019;104:802–14. 30982610 10.1016/j.ajhg.2019.03.002 PMC6507043

[btad139-B23] Li Z , Li X, Zhou H et al A framework for detecting noncoding rare-variant associations of large-scale whole-genome sequencing studies. Nat Methods 2022;19:1599–611. 36303018 10.1038/s41592-022-01640-x PMC10008172

[btad139-B24] Lin Y-C , Brooks JD, Bull SB et al Statistical power in covid-19 case-control host genomic study design. Genome Med 2020;12:1–8. 10.1186/s13073-020-00818-2 PMC7768597 33371892

[btad139-B25] Lyles RH , Lin H-M, Williamson JM. A practical approach to computing power for generalized linear models with nominal, count, or ordinal responses. Stat Med 2007;26:1632–48. 16817148 10.1002/sim.2617

[btad139-B26] Marees AT , de Kluiver H, Stringer S et al A tutorial on conducting genome-wide association studies: quality control and statistical analysis. Int J Methods Psychiatr Res 2018;27:e1608. 29484742 10.1002/mpr.1608 PMC6001694

[btad139-B27] Mayo O. A century of Hardy–Weinberg equilibrium. Twin Res Hum Genet 2008;11:249–56. 18498203 10.1375/twin.11.3.249

[btad139-B28] McCullagh P , Nelder JA. Generalized Linear Models. New York: Routledge, 2019.

[btad139-B29] Namjou B , Lingren T, Huang Y et al GWAS and enrichment analyses of non-alcoholic fatty liver disease identify new trait-associated genes and pathways across emerge network. BMC Med 2019;17:1–19. 31311600 10.1186/s12916-019-1364-z PMC6636057

[btad139-B30] Novikov I , Fund N, Freedman L. A modified approach to estimating sample size for simple logistic regression with one continuous covariate. Stat Med 2010;29:97–107. 19823978 10.1002/sim.3728

[btad139-B31] O’Brien R. Using the SAS system to perform power analyses for log-linear models. In: *Proceedings of the Eleventh Annual SAS Users Group International Conference*, pp. 778–82. Cary, NC: SAS Institute Inc., 1986.

[btad139-B32] Patil P , Peng RD, Leek JT. What should researchers expect when they replicate studies? a statistical view of replicability in psychological science. Perspect Psychol Sci 2016;11:539–44. 27474140 10.1177/1745691616646366 PMC4968573

[btad139-B33] Pirinen M , Donnelly P, Spencer CC. Including known covariates can reduce power to detect genetic effects in case-control studies. Nat Genet 2012;44:848–51. 22820511 10.1038/ng.2346

[btad139-B34] Plomin R , DeFries JC, Loehlin JC. Genotype-environment interaction and correlation in the analysis of human behavior. Psychol Bull 1977;84:309. 557211

[btad139-B35] Purcell S , Neale B, Todd-Brown K et al Plink: a tool set for whole-genome association and population-based linkage analyses. Am J Hum Genet 2007;81:559–75. 17701901 10.1086/519795 PMC1950838

[btad139-B36] Rao CR , Rao CR, Statistiker M et al Linear Statistical Inference and Its Applications. Vol. 2, New York: Wiley, 1973.

[btad139-B37] Rekaya R , Smith S, El Hamidi Hay NF et al Analysis of binary responses with outcome-specific misclassification probability in genome-wide association studies. Appl Clin Genet 2016;9:169. 27942229 10.2147/TACG.S122250 PMC5138056

[btad139-B38] Robinson LD , Jewell NP. Some surprising results about covariate adjustment in logistic regression models. Int Stat Rev 1991;59:227–40.

[btad139-B39] Scarr S , McCartney K. How people make their own environments: a theory of genotype → environment effects. Child Dev 1983;54:424–35. 6683622 10.1111/j.1467-8624.1983.tb03884.x

[btad139-B40] Self SG , Mauritsen RH. Power/sample size calculations for generalized linear models. Biometrics 1988;44:79–86.

[btad139-B41] Self SG , Mauritsen RH, Ohara J. Power calculations for likelihood ratio tests in generalized linear models. Biometrics 1992;48:31–9. 10.1111/j.0006-341x.2000.01192.x 11129478

[btad139-B42] Serfling RJ. Approximation Theorems of Mathematical Statistics. Vol. 162. New York: Wiley, 2009.

[btad139-B43] Shieh G. On power and sample size calculations for likelihood ratio tests in generalized linear models. Biometrics 2000;56:1192–6. 11129478 10.1111/j.0006-341x.2000.01192.x

[btad139-B44] Sjölander A , Greenland S. Ignoring the matching variables in cohort studies–when is it valid and why? Stat Med 2013;32:4696–708. 23761197 10.1002/sim.5879

[btad139-B45] Sudlow C , Gallacher J, Allen N et al UK Biobank: an open access resource for identifying the causes of a wide range of complex diseases of middle and old age. PLoS Med 2015;12:e1001779. 25826379 10.1371/journal.pmed.1001779 PMC4380465

[btad139-B46] Sun L , Bull SB. Reduction of selection bias in genomewide studies by resampling. Genet Epidemiol 2005;28:352–67. 15761913 10.1002/gepi.20068

[btad139-B47] Sun L , Dimitromanolakis A, Faye LL et al Br-squared: a practical solution to the winner’s curse in genome-wide scans. Hum Genet 2011;129:545–52. 21246217 10.1007/s00439-011-0948-2 PMC3074069

[btad139-B48] Turley P , Walters RK, Maghzian O et al Multi-trait analysis of genome-wide association summary statistics using MTAG. Nat Genet 2018;50:229–37. 29292387 10.1038/s41588-017-0009-4 PMC5805593

[btad139-B49] Wang Z , Sun L, Paterson AD. Major sex differences in allele frequencies for x chromosomal variants in both the 1000 genomes project and gnomAD. PLoS Genet 2022;18:e1010231. 35639794 10.1371/journal.pgen.1010231 PMC9187127

[btad139-B50] Weissbrod O , Flint J, Rosset S. Estimating SNP-based heritability and genetic correlation in case-control studies directly and with summary statistics. Am J Hum Genet 2018;103:89–99. 29979983 10.1016/j.ajhg.2018.06.002 PMC6035374

[btad139-B51] Whittemore AS. Sample size for logistic regression with small response probability. J Am Stat Assoc 1981;76:27–32.

[btad139-B52] Yang J , Benyamin B, McEvoy BP et al Common SNPs explain a large proportion of the heritability for human height. Nat Genet 2010;42:565–9. 20562875 10.1038/ng.608 PMC3232052

[btad139-B53] Yang J , Zeng J, Goddard ME et al Concepts, estimation and interpretation of SNP-based heritability. Nat Genet 2017;49:1304–10. 28854176 10.1038/ng.3941

[btad139-B54] Zhang Q , Yi GY. Genetic association studies with bivariate mixed responses subject to measurement error and misclassification. Stat Med 2020;39:3700–19. 32914420 10.1002/sim.8688

[btad139-B55] Zhong H , Prentice RL. Correcting “winner’s curse” in odds ratios from genomewide association findings for major complex human diseases. Genet Epidemiol 2010;34:78–91. 19639606 10.1002/gepi.20437 PMC2796696

[btad139-B56] Zhu Z , Zheng Z, Zhang F et al Causal associations between risk factors and common diseases inferred from GWAS summary data. Nat Commun 2018;9:1–12. 29335400 10.1038/s41467-017-02317-2 PMC5768719

[btad139-B57] Zöllner S , Pritchard JK. Overcoming the winner’s curse: estimating penetrance parameters from case-control data. Am J Hum Genet 2007;80:605–15. 17357068 10.1086/512821 PMC1852705

