# Supplementary material for: The hidden factor: accounting for covariate effects in power and sample size computation for a binary trait
Source: Bioinformatics. 2023 Mar 21;39(4):btad139. doi: 10.1093/bioinformatics/btad139 (PMC10070038; doi:10.1093/bioinformatics/btad139)
Supplement: btad139_Supplementary_Data [file btad139_supplementary_data.pdf]

Supplementary Material for “The hidden factor: accounting for covariate effect in power and sample size computation for a binary trait”

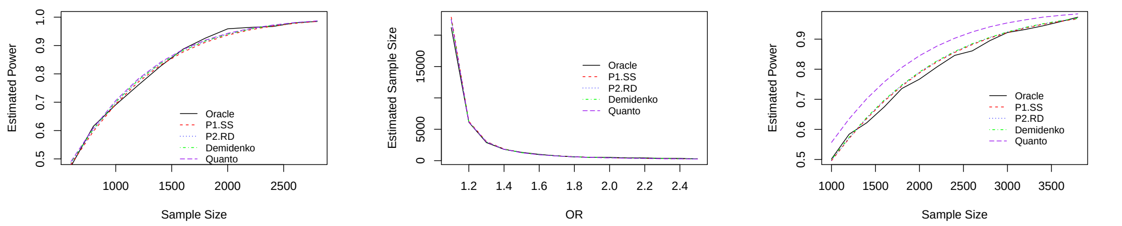

(a) S1: Retrospective without covariate

(b) S1: Retrospective without covariate

(c) S2: Prospective, binary covariate

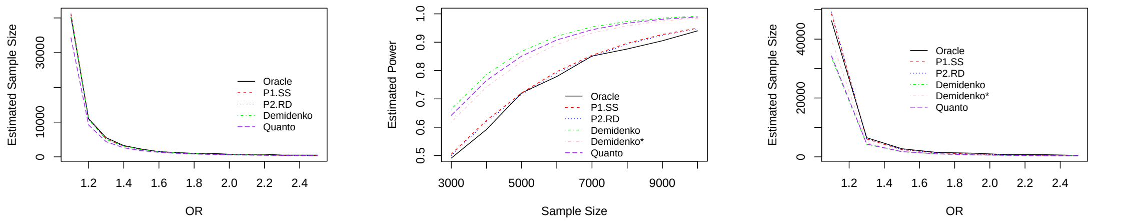

(d) S2: Prospective, binary covariate

(e) S3: Prospective, continuous covariate

(f) S3: Prospective, continuous covariate

Figure S1: Simulation results for the three scenarios considered in Section 4.1. Scenario 1 (S1) is the retrospective case-control sampling design without E. Scenario 2 (S2) and Scenario 3 (S3) are the prospective sampling design with, respectively, a binary and continuous covariate E. Figures (a), (c) and (e) on the left panel compare the power computation when β_G is fixed at log(1.5) (i.e. OR of 1.5, for (a-c)) or log(1.3) (for (e)), and Figures (b), (d) and (f) on the right panel compare the sample size computation to achieve power of 80% at the significance level of 0.05 across different $\exp(\beta_G)$. The red curves are for the ‘semi-simulation’ method (P1.SS in Section 3.2), blue curves for the ‘representative dataset’ method (P2.RD in Section 3.3), purple curves for `Quanto` of Gauderman (2002b), and green and pink curves for the method of Demidenko (2007); in S3, the method of Demidenko (2007) was implemented by dichotomizing E or without considering E). The black cures represent the oracle power and replication sample size estimated empirically.

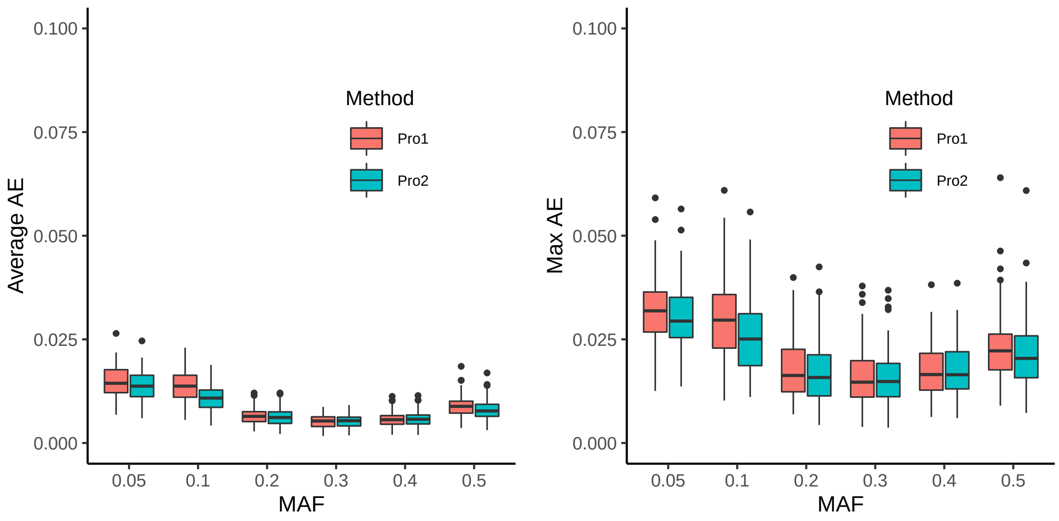

Figure S2: Additional results on the robustness of the proposed implementations to the choice of MAF. The simulation setting is the same as in Scenario 3 (S3) in Section 4.1. Given a MAF, the absolute error (AE) relative to the oracle power is computed at each choice of sample size (3,000 – 10,000). At each choice of sample size, oracle power is the empirical power computed using 1,000 independent replications. Then the average and maximum AE across all sample sizes are computed. We further repeat the procedure independently 100 times to obtain the boxplots of average (left) and maximum (right) AE at each MAF choice, for each of the proposed methods (P1:SS in red, P2:RD in blue).

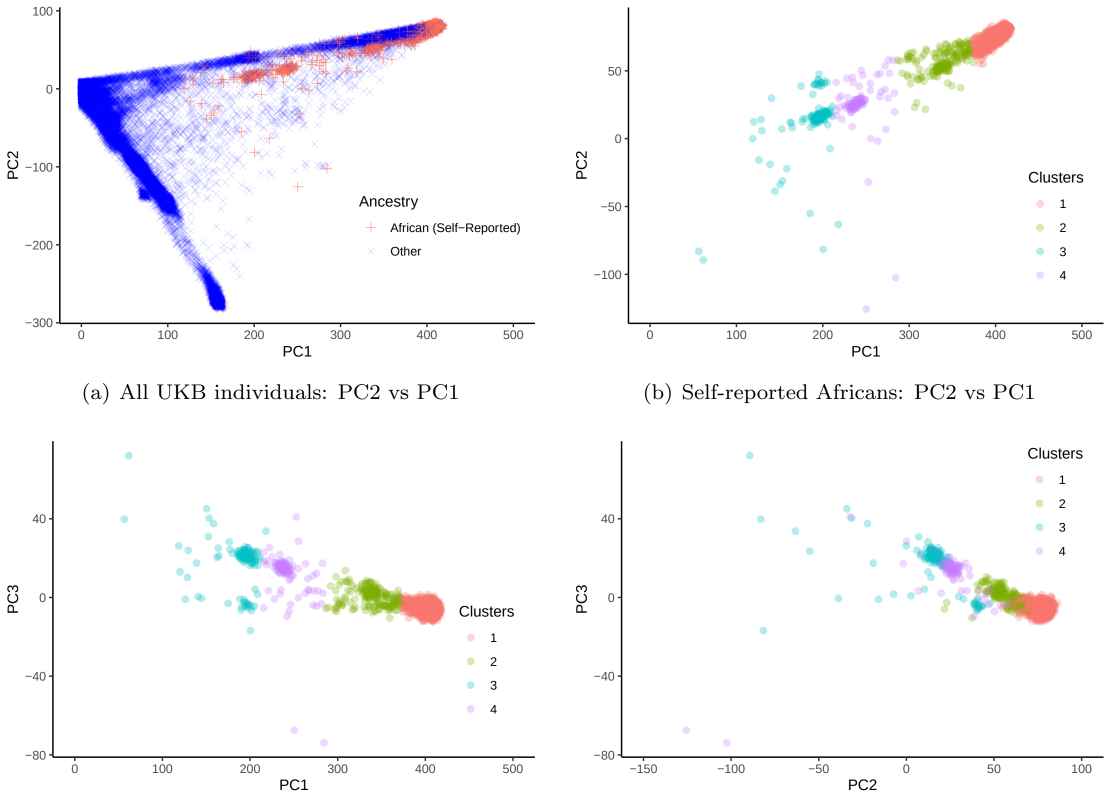

(a) All UKB individuals: PC2 vs PC1

(b) Self-reported Africans: PC2 vs PC1

(c) Self-reported Africans: PC3 vs PC1

(d) Self-reported Africans: PC3 vs PC2

Figure S3: Population principle components plots for (a) the whole UK Biobank sample, stratified by n = 3,460 self-reported African sample vs. Others, and (b)–(d) the self-reported African sample. In Figures (b)–(d), the four clusters were identified by a K-mean algorithm as discussed in Section 5. The GWAS shown in Figure S6 used the n = 2,510 individuals in Cluster 1, identified based on this PCA analysis.

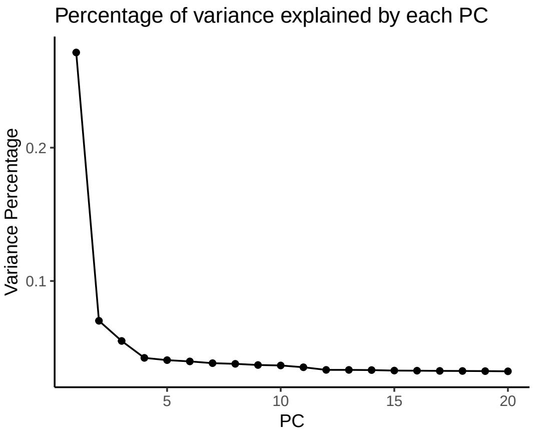

(a) Newly computed PCs: Elbow Plot

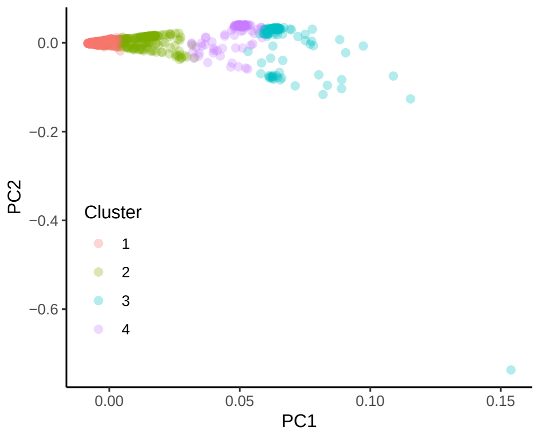

(b) Newly computed PCs: PC2 vs PC1

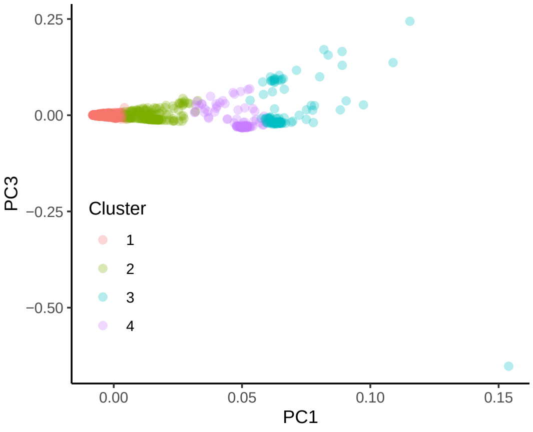

(c) Newly computed PCs: PC3 vs PC1

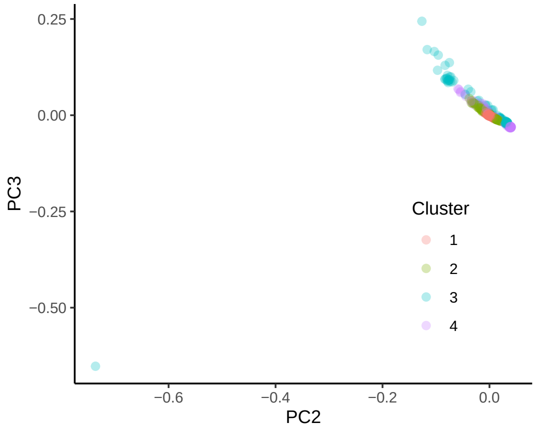

(d) Newly computed PCs: PC3 vs PC2

Figure S4: PCs plots of self-reported Africans using the PCs computed from self-reported Africans for section 5. Clusters identified by the K-means algorithm are shown in different colors in (b)-(d). Figure (a) is the elbow plot of newly computed PCs, which shows the first four PCs explained the majority of the total variance.

Hypertension							Blood Pressure						
SNP	CHR	REF	ALT	BetaG	MAF	P	SNP	CHR	REF	ALT	BetaG	MAF	P
rs17069257	8	T	C	0.333	0.194	1.15e-05	rs7158982	14	G	T	1.37	0.460	6.40e-06
rs9540255	13	A	G	0.274	0.420	9.43e-06	rs514400	18	C	T	1.88	0.162	5.93e-06
rs1648707	3	C	A	-0.279	0.411	8.17e-06	rs472771	1	G	A	-1.38	0.478	4.25e-06
rs163913	19	T	C	-0.399	0.156	5.21e-06	rs9313506	5	T	C	-1.83	0.206	9.17e-07
rs765528	6	C	A	-0.439	0.168	2.89e-07	rs596875	18	A	C	2.24	0.146	2.04e-07

Table S1: The GWAS summary statistics of the five selected SNPs in the analysis of the UK Biobank African sample (n = 2,510). As the GWAS did not identify any genome-wide significant SNPs, the selected ones are the five top-ranked SNPs that were closest to the genome-wide significance level. The baseline allele (REF) is the major allele, and the alternative allele (ALT) is the minor allele.

Hypertension							Blood Pressure						
SNP	CHR	REF	ALT	BetaG	MAF	P	SNP	CHR	REF	ALT	BetaG	MAF	P
rs805293	6	T	A	0.0347	0.452	4.94e-08	rs716428	4	C	T	-0.154	0.456	5.00e-08
rs975730	8	G	A	-0.0363	0.353	4.91e-08	rs9858542	3	G	A	-0.168	0.292	4.85e-08
rs2888877	7	C	T	-0.0437	0.197	4.90e-08	rs9268861	6	C	A	-0.187	0.210	4.77e-08
rs2736171	6	A	G	0.0358	0.361	4.90e-08	rs6066802	20	T	C	-0.174	0.265	4.74e-08
rs2417820	12	C	T	-0.0387	0.282	4.83e-08	rs58935064	14	C	A	-0.162	0.330	4.73e-08

Table S2: The GWAS summary statistics of the five selected SNPs in the analysis of the UK Biobank European sample (n = 276,682). As the GWAS identified many genome-wide significant SNPs, the selected ones are the five bottom-ranked genome-wide significant SNPs. The baseline allele (REF) is the major allele, and the alternative allele (ALT) is the minor allele.

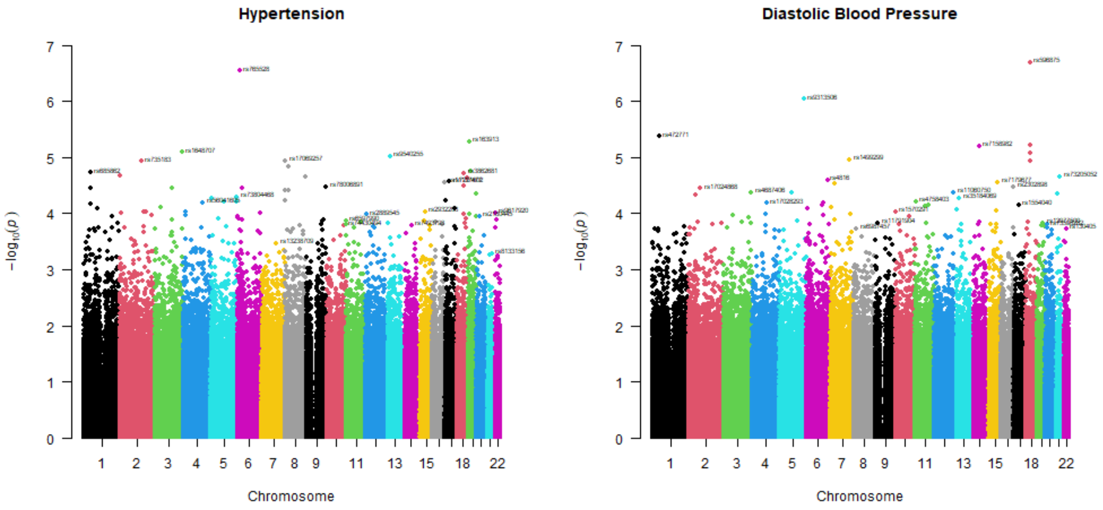

(a) The (binary) hypertension trait (b) The (continuous) blood pressure trait

Figure S5: GWAS Manhattan results for (a) the binary hypertension trait and (b) the continuous diastolic blood pressure trait, using the African sample (n = 2,510) identified through a PCA analysis of the self-identified African sample of the UK Biobank data as discussed in Section 5. The association analyses included age and sex as important covariates. No SNPs reached genome-wide significance level of 5e-8.

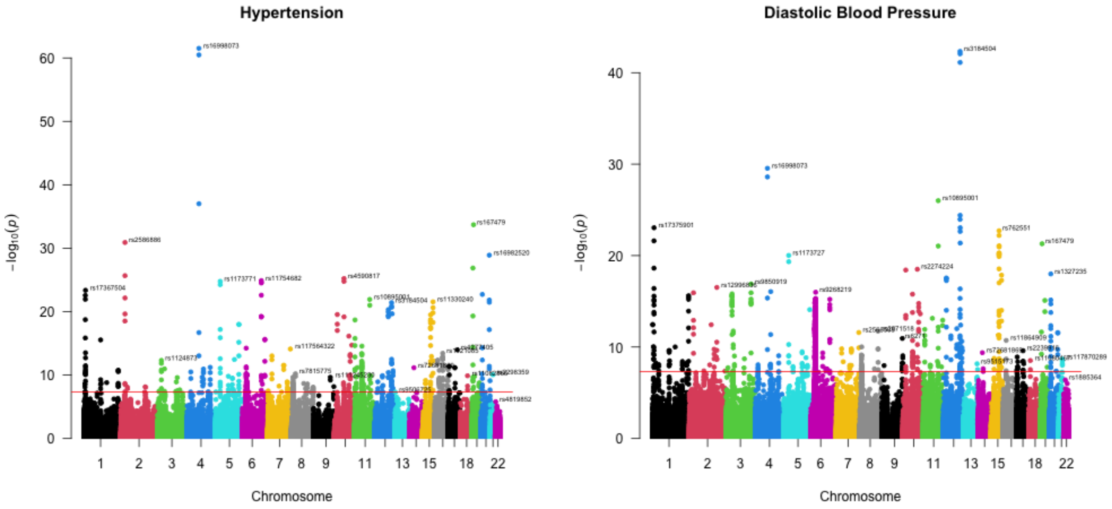

(a) The (binary) hypertension trait (b) The (continuous) blood pressure trait

Figure S6: GWAS Manhattan results for (a) the binary hypertension trait and (b) the continuous diastolic blood pressure trait, using the European sample (n = 276,682) of the UK Biobank data as discussed in Section 5. The association analyses included age and sex as important covariates.

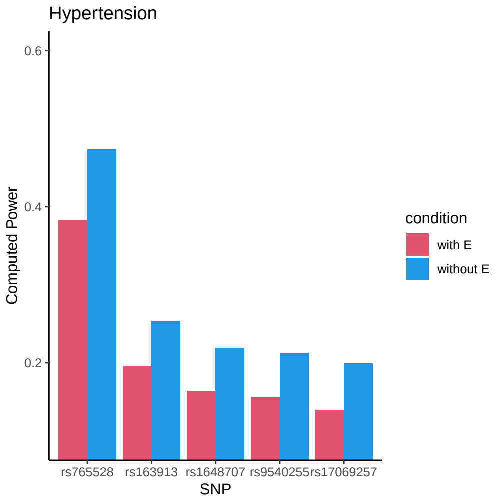

(a) *Overestimated* power for the (binary) trait if not accounting for E

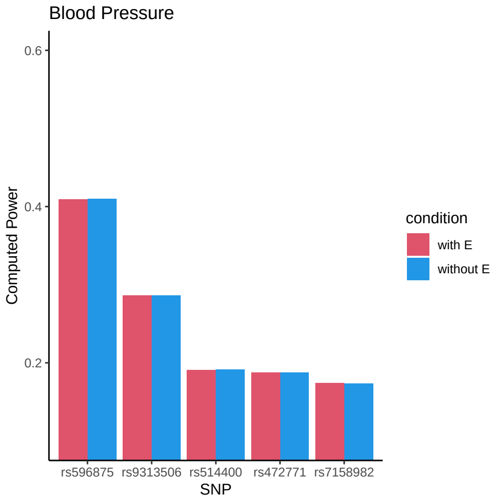

(b) Same power for the (continuous) trait

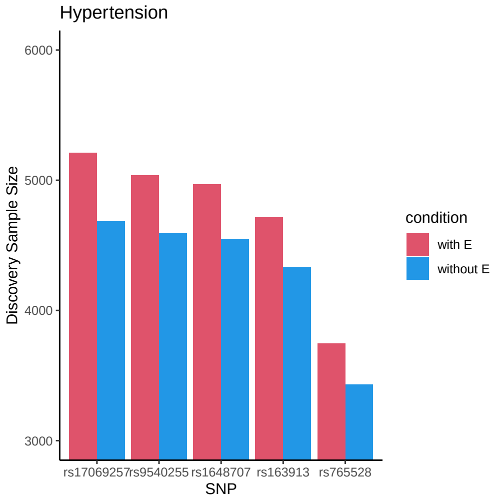

(c) *Underestimated* (Discovery) sample size for the (binary) trait if not accounting for E

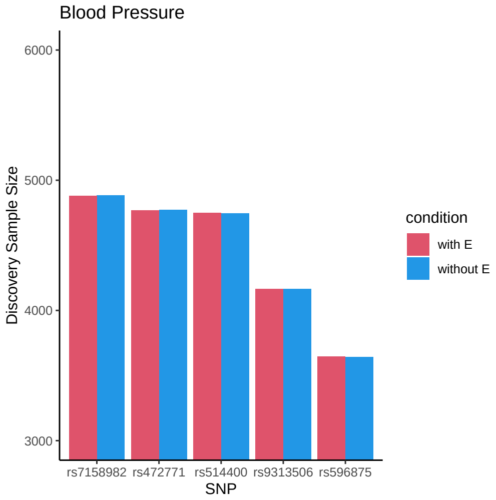

(d) Same (Discovery) sample size for the (continuous) trait

Figure S7: Powers (Figures (a) and (b)) and sample sizes (Figures (c)-(d)) estimation for study planning of the top five-ranked SNPs identified in GWAS of the binary hypertension trait (Figures (a), (c)) and the continuous diastolic blood pressure trait (Figures (b), (d)), using the African sample (n = 2,510) identified through a PCA analysis of the self-identified African sample of the UK Biobank data as discussed in Section 5. The genetic effects of these SNPs used for power and sample size computations are based on a standard GWAS, shown in Figure S6, where age and sex were included as important covariates. For (replication) study planning, the red bars are the computed power or sample size with adjustment for age and sex, and the blues bars are the values without explicitly considering age and sex. The two approaches do not have difference in power and sample size planning for the continuous blood pressure trait, as age and sex effects are incorporated through residual variance. In contrast, when analyzing a binary trait, the higher blue bars in Figure (a) show that ignoring covariate effects leads to *overestimated power* of our discovery study (at α = 5e-8); power for α = 0.05 is close to 100% as expected, thus not shown. The shorter blue bars in Figure (c) show that ignoring covariate effects leads to *underestimated discovery sample size* (for 80% power at α = 5e-8).

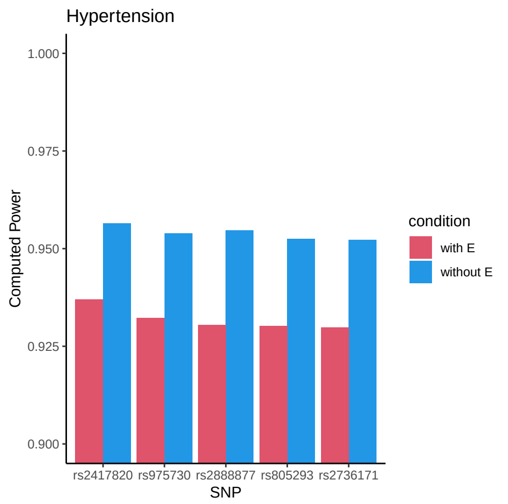

(a) *Overestimated* power for the (binary) trait if not accounting for E

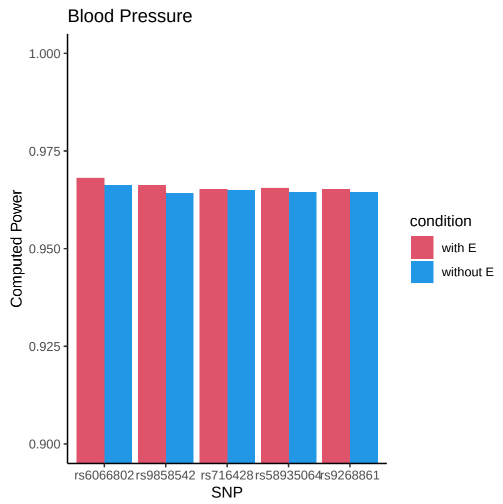

(b) Same power for the (continuous) trait

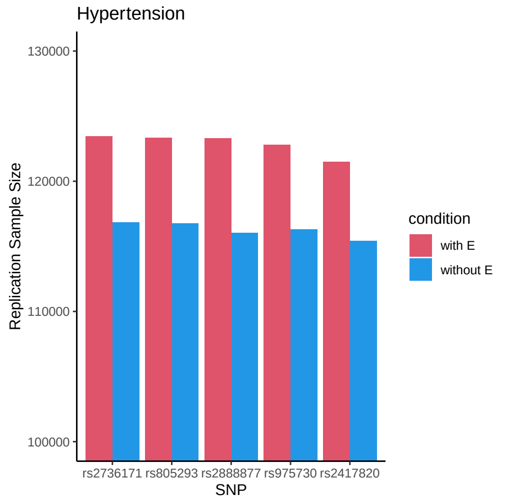

(c) *Underestimated* (Replication) sample size for the (binary) trait if not accounting for E

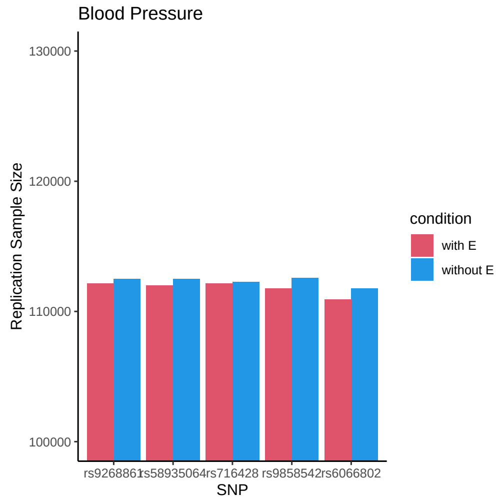

(d) Same (Replication) sample size for the (continuous) trait

Figure S8: Powers (Figures (a) and (b)) and replication sample sizes (Figures (c)-(d)) estimation for study planning of the five bottom-ranked genome-wide significant SNPs identified in GWAS of the binary hypertension trait (Figures (a), (c)) and the continuous diastolic blood pressure trait (Figures (b), (d)), using the European sample (n = 276,682) confirmed using genetic data (Field ID: 22006). The genetic effects of these SNPs used for power and sample size computations are based on a standard GWAS, shown in Figure S6, where age and sex were included as important covariates. For (replication) study planning, the red bars are the computed power or sample size with adjustment for age and sex, and the blues bars are the values without explicitly considering age and sex. The two approaches do not have difference in power and sample size planning for the continuous blood pressure trait, as age and sex effects are incorporated through residual variance. In contrast, when analyzing a binary trait, the higher blue bars in Figure (a) show that ignoring covariate effects leads to overestimated power of our discovery study (at α = 5e-8); power for $\alpha = 0.05$ is close to 100% as expected, thus not shown. The shorter blue bars in Figure (c) show that ignoring covariate effects leads to underestimated replication sample size (for 80% power at α = 5e-3).
